# Research highlights, *The Plant Genome*, Volume 14, Issue 1

**DOI:** 10.1002/tpg2.20093

**Published:** 2021-03-14

## 1 UNDERSTANDING NLRS FOR BETTER RESISTANCE

Understanding the evolutionary history and variations in NLR disease resistance genes is important to protect Brassica vegetable and oilseed crops from damage caused by disease. Zhang et al. (https://doi.org/10.1002/tpg2.20060) show that duplications at the genome and sub‐genome levels, selection from pathogens and recombination within Brassica populations have shaped the highly duplicated and diverse NLR patterns in Brassica genomes. The number and clustering pattern of NLR genes in Brassica vegetable and canola genomes were compared to understand the evolutionary patterns of the NLR genes. Studies on the identification of disease resistance genes through comparative approaches were highlighted to address the importance of NLR duplication and diversification on strengthening disease resistance in Brassicas. This review is crucial to help gain insights into their function and inform the identification of resistance genes for breeding of resistant lines.

## 2 GWAS: PAST, PRESENT, AND FUTURE

Genome‐wide association studies (GWAS) are a powerful tool for studying complex traits in crops. Tibbs Cortes et al. (https://doi.org/10.1002/tpg2.20077) review advancements in GWAS methods that build on the mixed linear model framework to improve both computational speed and statistical power. Current challenges in GWAS will provide new opportunities for future research, as GWAS can lead to increased understanding of trait biology as well as practical guidelines for genomic selection and genome editing. Therefore, continued GWAS method development and implementation will have a broad impact on plant biology and crop improvement.

## 3 A MAJOR QTL RESISTANT TO *PUCCINIA POLYSORA* IN MAIZE

Southern corn rust (SCR) caused by *Puccinia polysora* Underw is a prevalent foliar disease in maize. Resistance to SCR mainly relies on the discovery of major quantitative trait loci (QTLs). Lv et al. (https://doi.org/10.1002/tpg2.20062) report a major QTL, *RppCML496*, resistant to southern corn rust on the short arm of chromosome 10. Using a fine mapping strategy, *RppCML496* was delimited to an interval of 128 Kb. Genome mining of this region suggests two candidate genes, and a NBS‐LRR gene is the promising one for *RppCML496* against SCR. The tightly linked molecular markers developed in this study can be used for molecular breeding of resistance to SCR in maize.

## 4 IDENTIFYING SOYBEAN NOVEL *RPS* LOCI

*Phytophthora sojae* causes Phytophthora root and stem rot of soybean and has been primarily managed through deployment of qualitative *Resistance to P. sojae* (*Rps*) genes. Due to the complex nature of *P. sojae* populations, identification of more novel *Rps* genes is needed. Van et al. (https://doi.org/10.1002/tpg2.20063) conducted genome‐wide association (GWA) analyses using phenotypic and genotypic data of 16 plant introduction (PI) panels. They identified two, two, six, and seven novel *Rps* loci with Panels 1 (448 *Glycine max*), 2 (520 *G. soja*), 3 (429 *G. max*), and 4 (460 *G. max*), respectively, and 58 novel *Rps* loci with Panels 5–16 (376 *G. max*). Genetic and phenotypic dissection of these loci may help the characterization of novel *Rps* genes that can be used for development of new soybean cultivars with effective resistance against diverse *P. sojae* populations.

## 5 GENOMIC PREDICTION FEASIBLE IN SAFFLOWER

The oilseed crop safflower is enjoying renewed interests but has seen limited breeding activity. Incorporating genomic prediction into safflower germplasm evaluation and future breeding programs could increase safflower breeding efficiency dramatically. Zhao et al. (https://doi.org/10.1002/tpg2.20064) used a safflower genebank collection as the training population and genomic prediction accuracies were moderate to high for grain yield and its related traits with linear mixed models. Diverse responses to the local environments and moderate to high genomic heritability were observed for all traits. Quality control thresholds for genotyping‐by‐sequencing SNP data affected genomic heritability estimates, but genomic prediction accuracy was more robust.

## 6 GWAS FOR STRIPE RUST RESISTANCE

Stripe rust of wheat is one of the most devastating disease of bread wheat and the new pathotypes of stripe rust are currently widespread from Asia to Europe, Africa, and Australia, threatening wheat yields at a global level. Tehseen et al. (https://doi.org/10.1002/tpg2.20066) emphasized the prospects of taking advantage of high genetic diversity in bread wheat landraces preserved at the gene banks. 600 landraces were evaluated for seedling and adult plant resistance against the *PstS2* and *Warrior* pathotypes of stripe rust. The study identified 47 significant SNP markers for both seedling stage and adult plant resistance. Three genomic regions (*QYr.1D_APR, QYr.3A_seedling* and *QYr.7D_seedling*) identified did not correspond to any previously reported *Yr* genes or QTL, suggesting new genomic regions for stripe rust resistance. The identified resistance QTL could serve as reliable breeding tools for future wheat breeding programs addressing stripe rust.

## 7 IMPROVING STARCH QUALITY IN SORGHUM

Landraces of sorghum exhibit variation in food quality traits but our understanding of the genes controlling these traits in sorghum is limited. In this study, Griebel et al. (https://doi.org/10.1002/tpg2.20067) screened a large collection of diverse sorghum breeding lines and sorghum conversion (SC) lines for variation in alkali spreading value (ASV) and discovered accessions, especially Nandyal types with origins in India, with unique starch quality characteristics. Genomic analyses linked these characteristics with variations in genes involved in starch biosynthesis. These genes and the alleles represented in these accessions could be valuable for modifying starch to develop new food products.

## 8 HOMOEOLOGOUS COPY NUMBER VARIATIONS IN WHEAT

Wheat is an allohexaploid species originating from two successive and recent rounds of hybridization between three diploid species with very similar genomes. Notwithstanding, most of the genes are not present in three homoeologous copies. Juery et al. (https://doi.org/10.1002/tpg2.20069) report on the analysis of genes being in two, three or four homoeologous copies in the wheat genome. By analyzing their distribution, conservation, function, expression and epigenetic profiles, they show that triads, i.e. genes in three copies, mainly correspond to housekeeping genes and are part of the core genome, while dyads and tetrads, i.e. genes having undergone a single copy deletion or duplication belong to the dispensable genome of wheat. They also suggest the differences observed between the subgenomes are likely related to two successive and ongoing waves of post‐polyploid diploidization and that, unlike most of the allopolyploid species, subgenome dominance and biased fractionation are absent in hexaploid wheat.

## 9 GENETIC MAPPING OF *HELICOVERPA* COMPONENT TRAITS IN CHICKPEA

*Helicoverpa armigera* (Hübner), a major pest, damages chickpea crops and causes substantial economic and yield losses. Barmukh et al. (https://doi.org/10.1002/tpg2.20071) report a genetic map for the *H. armigera* resistance component traits to identify quantitative trait loci (QTLs) based on a comprehensive analysis of the phenotyping and genotyping data on an interspecific bi‐parental mapping population. Some of these QTLs were found linked with previously described genes, known to modulate resistance against lepidopteran insects in crop plants. Interestingly, *H. armigera* resistance component traits in chickpea seems to be controlled by a limited number of genes with large effects.

## 10 DRAFT PHASED ASSEMBLY OF CASCADE HOP

Genomic resources for hop have been hindered because of the large size and complexity of the hop genome. With advances in sequencing and assembly technology, a deeper understanding of the biology and evolution of the hop genome has become attainable. Padgitt‐Cobb et al. (https://doi.org/10.1002/tpg2.20072) provide a draft phased assembly of the diploid genome, containing a partially‐resolved assembly for each haplotype, resulting in a primary and associate assembly. They developed an approach to further refine the diploid‐aware assembly by improving the detection of duplicated primary contigs. They provide an updated assessment of repeat content in the genome, and identified conserved regions of co‐linear genes between hop and hemp, as well as a gene with strong homology to cannabidiolic acid synthase that is expressed in multiple tissues in hop, with highest expression occurring in leaf tissue. The improved and more‐comprehensive assembly and annotations will inform hop breeding efforts and broaden the potential comparative genomic studies that can be performed with other members of the Cannabaceae.

## 11 AFRICAN BERMUDAGRASS GENOME IS MAPPED

African bermudagrass has been artificially crossed to common bermudagrass in the creation of high quality interspecific F1 hybrid turf cultivars for use on sports fields, golf courses and home lawns. Yu et al. (https://doi.org/10.1002/tpg2.20073) report the first high‐density genetic map in African bermudagrass. Using genotyping‐by‐sequencing, they discovered and used single nucleotide polymorphism markers in the genetic mapping work. They identified four genomic regions associated with establishment rate in QTL analysis. The findings provide valuable information for further investigation of the genome and development of new cultivars.

## 12 GENOMICS‐ASSISTED BREEDING IN RICE

Food crop productions and nutritional properties will be consistently and negatively affected by increasing temperature due to climate change. Yadav et al. (https://doi.org/10.1002/tpg2.20074) performed a genomic‐assisted breeding strategy to assemble multiple quantitative trait loci (QTLs) and genes and develop climate‐smart, superior grain quality rice with higher grain yield. The developed lines possessing genes and QTLs for different stresses are free from undesirable linkage drags and can be used to introgress multiple genes and QTL in other backgrounds with ease and with higher chances of success or can be released as varieties.

## 13 DROUGHT TOLERANT AND HIGH‐YIELDING CHICKPEA LINES THROUGH MOLECULAR BREEDING

With an aim of developing drought tolerant chickpea varieties, Bharadwaj et al. (https://doi.org/10.1002/tpg2.20076) report introgression of “*QTL‐hotspot*” region for drought tolerance into three chickpea cultivars following molecular breeding. This study reports development of many introgression lines with higher yield performance in rainfed conditions. Multi‐location evaluation of several of these lines through the rigorous varietal identification and release pipeline already released the Pusa Chickpea 10216 variety for commercial cultivation in India. This study, in summary, is a success story of genomics‐assisted breeding to deliver better varieties for addressing the climate change issues in chickpea.

## 14 STRUCTURAL REARRANGEMENTS IN WHEAT (1BS) ‐ RYE (1RS) RECOMBINANT CHROMOSOMES AFFECT GENE DOSAGE AND ROOT LENGTH

Wheat is an important global crop grown in different soils around the world.  A better understanding of the genes regulating different root architectures is important to develop wheat varieties better adapted to different types of soils. In this study, Gabay et al. (https://doi.org/10.1002/tpg2.20079) identified a small, duplicated chromosome region affecting wheat root length, which was previously associated with differences in grain yield under drought. Within this region, the authors identified 14 genes expressed in roots that are candidates for the regulation of wheat root length and architecture.

## 15 MILLET SPREAD IN FOUR GENETIC GROUPS

The genetic diversity in cultivated plants records the history of their origins, spread, and adaptation to novel environments. Hunt et al. (https://doi.org/10.1002/tpg2.20081) found that foxtail millet in Eurasia and Africa divides into four genetic groups. These four groups diverged following a single domestication from green foxtail. The groups showed distinct patterns reflecting foxtail spread west, east, and south from China, inferred from both genomic and archaeobotanical data. Nine genes show evidence that different groups evolved specific adaptations to high‐latitude and high‐altitude environments as millet spread.

## 16 CATEGORIZING SOUTHEASTERN U.S. STRIPE RUST RESISTANCE

Stripe rust is a fungal pathogen of wheat that can lead to severe economic losses for growers. Many stripe rust races and resistance genes have been cataloged around the globe, though no systematic survey of stripe rust resistance present in the soft red winter wheat grown in the eastern United States has been performed. Ward et al. (https://doi.org/10.1002/tpg2.20082) investigated the stripe rust resistance loci most important in soft red winter wheat by performing a genome‐wide association study using both historical and contemporary breeding material. They found that three regions on chromosomes 2A, 3B, and 4B accounted for most of the stripe rust resistance observed. The 4B region was the most important for resistance though it produced unstable effects across the testing panels. The 2A region was introduced to regional germplasm in the early 2000’s, but it produces more modest resistance in modern breeding lines.

## 17 *MDPAE10* AFFECTS APPLE FRUIT SHELF LIFE

Room‐temperature shelf life is a key factor in fresh market apple (*Malus domestica* Borkh.) quality and commercial value. Flesh firmness and crispness are important texture attributes for fruit shelf life. A number of cell wall metabolism‐related genes, *MdPG1*, *MdPME*, *MdPel*, and *MdExp7* have been reported to promote the degradation of fruit cell walls and therefore accelerate flesh softening (Costa et al., 2008; Zhang et al., 2018; Longhi et al., 2013). In this study, Wu et al. (https://doi.org/10.1002/tpg2.20084) detected segregation of flesh firmness or crispness traits in 1,273 F1 plants of ‘Zisai Pearl’ × ‘Golden Delicious’. Thirteen quantitative trait loci (QTLs), including three major ones, were identified on chromosome 03, 08, and 16. A candidate gene encoding pectin acetylesterase, *MdPAE10*, from the QTL Z16.1 negatively affected fruit shelf life.

## 18 DISCOVERY OF VARIATION FOR VITAMIN A IN TOMATO

β‐carotene is a plant pigment with provitamin A activity important for human nutrition. Orchard et al. (https://doi.org/10.1002/tpg2.20085) discovered genetic variation in databases of DNA sequence for tomato, and accelerated breeding techniques were used to identify new versions with increased vitamin levels. Wild tomato relatives were an important source of increased β‐carotene. The important sequence variation was in the switch, or promoter, which turns a gene controlling pigment levels on in fruit.

